# *Lactobacillus helveticus* EL2006H cell-free supernatant enhances growth variables in *Zea mays* (maize), *Glycine max* L. Merill (soybean) and *Solanum tuberosum* (potato) exposed to NaCl stress

**DOI:** 10.3389/fmicb.2022.1075633

**Published:** 2023-01-10

**Authors:** Judith Naamala, Levini A. Msimbira, Sowmyalakshmi Subramanian, Donald L. Smith

**Affiliations:** Department of Plant Science, McGill University, Montreal, QC, Canada

**Keywords:** cell-free supernatant, *Lactobacillus helveticus*, salinity stress, germination, radicle length, plant growth

## Abstract

Plant growth promoting microorganisms and their derived compounds, such as cell-free supernatant (CFS), enhance plant growth under stressed and non stressed conditions. Such technology is sustainable and environmentally friendly, which is desirable amidst the climate change threat. The current study evaluated the effect of CFS obtained from *Lactobacillus helveticus* EL2006H on its ability to enhance mean percentage germination and mean radicle length of corn and soybean, as well as growth parameters of potato, using treatment formulations that consisted of 0.2 and 1.0% [v/v] *L. helveticus* EL2006H CFS concentrations and 100 mM NaCl and 150 mM NaCl levels. Results show that treatment with 100 mM NaCl lowered percentage germination of corn by 52.63%, at 72 h, and soybean by 50%, at 48 h. Treatment with 100 NaCl +0.2% EL2006H enhanced percentage germination of soybean by 44.37%, at 48 h, in comparison to that of the 100 mM NaCl control. One hundred mM NaCl lowered radicle length of corn and soybean by 38.58 and 36.43%, respectively. Treatment with 100 Mm NaCl +1.0% EL2006H significantly increased radicle length of corn by 23.04%. Treatment with 100 mM NaCl +0.2% EL2006H significantly increased photosynthetic rate, leaf greenness and fresh weight of potato. Increasing NaCl concentration to 150 NaCl lowered the effectiveness of the 0.2% EL2006H CFS on the same growth variables of potato. In general, the lower CFS concentration of 0.2% was more efficient at enhancing germination in soybean while the higher concentration of 1.0% was more efficient at enhancing radicle length of corn. There was an observed variation in the effectiveness of *L. helveticus* EL2006H CFS across the different CFS concentrations, NaCl levels and crop species studied. In conclusion, based on findings of this study, CFS obtained from *L. helveticus* can be used as a bio stimulant to enhance growth of corn, soybean and potato. However, further studies need to be conducted, for validation, especially under field conditions, for commercial application.

## 1. Introduction

Plant growth promoting microorganisms (PGPM) live in close association with their host plants, forming a holobiont (Hartmann et al., 2014; Lyu et al., 2021); these relationships have existed for at least half a billion years (Knack et al., 2015). A plant’s exudates into its surroundings are a major determinant of the phytomicrobiome composition in its rhizosphere (Zhang et al., 2017). The association between PGPM and their host plants can enhance the latter’s growth and development, through mechanisms such as biostimulation, mitigation of abiotic stress effects, bioremediation and biocontrol (Glick, 2012; Ahemad and Kibret, 2014; Backer et al., 2018; Naamala and Smith, 2020), in a sustainable and environmentally friendly manner (Naamala and Smith, 2021a,b). PGPM and their derived compounds can be utilised in singular or consortium forms, results varying in such a way that some strains may be more effective when applied as single cells while others, in a consortium (Giassi et al., 2016; Subramanian et al., 2016a,b; Shah et al., 2022). *Lactobacillus helveticus* is a gram positive facultative anaerobic lactic acid bacterium (LAB) that is mostly known for its role in the food processing industry. Use of *L. helveticus* in plant agriculture, especially as biostimulants is not widely documented although the use of members of the genera *Lactobacillus* in crop production, as biostimulants and biocontrol agents, among other uses, has been practiced (Hamed et al., 2011). Members of the genus *Lactobacillus* are endophytic to a variety of plants species (Baffoni et al., 2015; Minervini et al., 2015; Lamont et al., 2017) while others have been isolated from the rhizosphere of plants. Examples of LAB species that have been used in plant agriculture include *Lactobacillus acidophilus*, *Lactobacillus plantarum*, *Lactobacillus rhamnosus* and *L. helveticus* (Hamed et al., 2011; Caballero et al., 2020; Msimbira et al., 2022). LAB play an important role in fermentation of organic matter to form organic fertilisers used in crop production (Lamont et al., 2017; Caballero et al., 2020). Lactic acid, a by-product of LAB has been reported to enhance plant growth (Rodríguez-Morgado et al., 2017). LAB strains have been reported to solubilise phosphate, produce siderophores (Shrestha et al., 2014; Giassi et al., 2016), produce antimicrobial compounds (Stoyanova et al., 2012), produce phytohormones such as indole −3- acetic acid [IAA] (Shrestha et al., 2014; Giassi et al., 2016) and enhance systemic acquired resistance (Hamed et al., 2011), all of which are desirable characteristics of PGPM.

Microbes, such as bacteria and fungi, exude into their growth environment, secondary metabolites such as hormones, enzymes, organic acids, bacteriocins, oligopolysaccharides and siderophores, among others (Piechulla et al., 2017; Schulz-Bohm et al., 2017; Lemfack et al., 2018). In laboratory-based experiments, such metabolites are exuded into the microbe’s growth medium. Recent studies have focused on the possibility that such metabolite rich media, also known as cell-free supernatant (CFS) can enhance plant growth without the presence of microbial cells. Experimentation with CFSs has previously yielded interesting results, showing that such CFSs can enhance the growth of different crop species, such as corn, soybean, canola and tomato, at germination and seedling stages (Msimbira et al., 2022; Naamala et al., 2022, Shah et al., 2022). Earlier studies went further and extracted bioactive compounds from the CFS, resulting in the discovery of compounds such as lipo-chitooligosaccharide (LCO) from *Bradyrhizobium japonicum* CFS and thuricin17 from *Bacillus thuringiensis* NEB17 CFS (Prithiviraj et al., 2003; Gray et al., 2006a,b; Lee et al., 2009), which are already on the market as plant growth promoting biostimulants. Both compounds have shown efficacy in enhancing plant growth under growth chamber and field conditions (Subramanian et al., 2016a,b; Arunachalam et al., 2018). They were reported to enhance plant growth under normal and salt-stress conditions (Subramanian et al., 2016a,b). It has been reported that the nature of metabolites exuded vary with varying conditions of growth media in which the microbe is growing (Subramanian et al., 2021). For instance, the metabolic profile of CFS of a microbe grown under ideal conditions may significantly vary from that of the same microbe exposed to some level of stress, such as low pH or high salinity levels. Not all metabolites exuded in growth media enhance plant growth, although some are the bioactive ingredients that do so. The effectiveness of a bioactive metabolite/CFS also varies across plant species, concentration of the compound, level of stress to which a plant is exposed and growth stage of the plant (Msimbira et al., 2022; Naamala et al., 2022; Shah et al., 2022). This, in a sense, complicates the process of discovering novel microbe derived plant growth promoting compounds as it may require trials on several crop species under different growth conditions, at different concentrations, before bioactivity may be detected. The seemingly long and possibly complicated process is however potentially worth the effort since microbial derived compounds can overcome some issues associated with using microbial cells as inoculants if they are produced in fairly large quantities and are economical to isolate for application. For instance, compounds are less prone to diminished effects under harsh field conditions, are generally required in low concentrations and are easier to store compared to live microbial cells (Naamala and Smith, 2021a).

Salinity stress is a major abiotic stress of agricultural crops, resulting in decreased yield quantity and quality which subsequently causes economic losses estimated at US$ 12 billion per year (FAO, 2020). It affects leaf area, chlorophyll content, plant vigour, plant height, rootlength, plant dry matter, nutrient, metabolite and protein contents, can delay plant development and at severe stress levels may lead to plant death (Bistgani et al., 2019; Garcia et al., 2019). Unfortunately, with current climate change projections, reduced rainfall, excess and improper application of inorganic fertilisers and other chemicals, arable land affected by salinity stress is projected to increase by 50% by 2050 (Jamil et al., 2011). PGPM and their derived compounds can mitigate the effect of salt stress on plants, hence, allowing better growth, yield quality and yield quantity, in salt affected fields (Naamala and Smith, 2021b). The aim of this study therefore was to elucidate the ability of *L. helveticus* EL2006H CFS to enhance germination and radicle length of corn and soybean, and growth parameters of potato, under saline conditions. Results of the study will be a baseline for further studies, with a possibility of isolating and identifying bioactive compounds. This study is part of a broader study that is studying CFSs of the EVL Inc., consortium strains, which comprises *L. helveticus* EL2006H and four other microbial species, in an effort to improve the product and or come up with new product combinations.

## 2. Materials and methods

### 2.1. Obtaining microbial CFS

*L. helveticus* EL2006H was cultured in De man, Rogosa and Sharpe (MRS) medium at pH 7.0, and incubated for 48 h, at 120 rpm and 37°C. At 48 h, the microbial culture was centrifuged for 10 min, at 10,000 rpm and 4°C, to pellet the microbial cells and separate them from the CFS (Gray et al., 2006a,b; Subramanian et al., 2021). The CFS was further filtered using 0.22 μm nylon filters to remove any microbial cells that could have remained after centrifugation. The obtained CFS was then used in the formulation of treatments used in the study.

### 2.2. Formulation of treatments

Treatments were formulated by mixing known quantities of distilled water, NaCl and CFS. Two NaCl levels, (100 mM NaCl and 150 mM NaCl) and two CFS levels (1.0 and 0.20% [v/v]) were used in the mixtures to formulate treatments. The two CFS concentrations were chosen because they exhibited positive results with *Bacillus amyloliquefaciens* EB2003 CFS, in our previous study (Naamala et al., 2022). 0, 100 and 150 mM NaCl, with no addition of CFS were used as negative controls. In addition, for each microbial CFS concentration, a similar concentration of microbial growth medium (not inoculated with microbe), was used to formulate positive controls. A treatment name ending in MRS or EL2006H implies that MRS medium and *L. helveticus* EL2006H CFSs were used, respectively.

### 2.3. Set up of germination and radicle length experiments

The germination experiments were carried out in a phytorium located at the Macdonald Campus of McGill University, Sainte Anne de Bellevue, Quebec, Canada. Soybean (cultivar P0962X) and corn (Hybrid 25 M75) were used for the study. The two crop species were chosen because they are widely consumed in Canada and the world over. In our previous study, the two species’ germination and radicle lengths were stimulated by *B. amyloliquefaciens* CFS. The following treatments were used for the study: 0 mM NaCl (control), 100 mM NaCl (control), 100 mM NaCl +0.2% MRS (control), 100 mM NaCl +0.2% EL2006H, 100 mM NaCl +1.0% MRS (control) and 100 mM NaCl +1.0% EL2006H. Treatments with 0.2% EL2006H and 1.0% EL2006H, with their corresponding controls, were studied separately. It should also be noted that each crop was studied separately. Therefore, because each treatment, within each crop species, was studied in a separate experiment and the data obtained analysed separately, a completely randomised design (CRD) was used for each experiment, to randomly apply experimental units to treatments. For each experiment, ten seeds of the crop species under study were surface sterilized using 2% sodium hypochlorite, for 2 min, rinsed with 5 changes of sterilized distilled water and placed on petri-plates (Cat. no. 431760, sterile 100 × 15 mm polystyrene Petri dish, Fisher Scientific Co., Whitby, ON, Canada), lined with filter paper (09-795D, QualitativeP8, porosity coarse, Fisher Scientific Co., Pittsburg, PA, United States). Petri plates with seeds then randomly received the treatments with ten replicates per treatment, hence, 40 samples per experiment. The Petri plates were then sealed with parafilm and incubated for 7 days in the dark, at 25°C. Total number of germinated seeds per plate was recorded at 24 h intervals, for 72 h, as a percentage of the total number of seeds in the plate. i.e., (x/10)∗100, where X is the total number of germinated seeds per petri plate. After 7 days, radicle length was measured, in centimeters (cm). For each replicate, radicle length for all the germinated seeds, was summed, to obtain total radicle length of germinated seeds per plate. Each experiment was repeated twice. Percentage germination data for each time interval (24, 48, and 72 h) were analyzed separately.

### 2.4. Set up of greenhouse experiment

Potato cultivar goldrush was used for the study. Potato is grown and widely consumed in Canada, with a sizable fresh market area of production in Quebec. EVL Inc., the source of the bacterial strains in collaboration with SynAgri, focus on cultivation of potato cultivars. Hence this part of the study was focused on potato’s response to treatment with the CFS, under greenhouse conditions. Treatments used for this experiment were: 0 mM NaCl (control), 100 mM NaCl (control), 100 mM NaCl +0.2% MRS (control), 100 mM NaCl +0.2% EL2006H, 150 mM NaCl (control), 150 mM NaCl +0.2% MRS (control) and 150 mM NaCl +0.2% EL2006H. Twelve L pots were filled with G7 growth medium were used for plant growth. The rooting medium in each pot was fully saturated with water before sowing one potato seed per pot. At emergence, pots were allocated to treatments following a CRD, with four replicates per treatment, hence, a total of 28 samples. The experiment was repeated twice. A number of excess pots were sown with seed so that on the day of treatment application, only pots with seeds that emerged on the same day were applied to treatments, to minimize initial variation. Two L of treatment were applied twice a week, per pot, for 4 weeks after emergence, at which time harvesting was conducted. Data on variables: greenness, photosynthetic rate, leaf area, plant height and plant fresh weight were taken. Leaf greenness was measured in SPAD units, using a SPAD-502 chlorophyll meter at 3 weeks after treatment application. Greenness of ten leaves was randomly measured and average greenness recorded. Photosynthetic rate was measured in μmol CO_2_ m^−2^ S^−1^ using a LI-COR 6400 portable photosynthesis meter (Lincoln, NE, United States), and recorded, 3 weeks after treatment application. Plant height was measured using a meter ruler, first, at emergence, just before the first application of treatments and 3 weeks after emergence. The difference in height was then recorded. Fresh weight was measured in grams, using a weighing scale balance (ME4001E, CH), 4 weeks after emergence. Leaf area was measured in cm^2^, using a leaf area meter (LI-3100 C, Lincoln, NE, United States), 4 weeks after emergence.

### 2.5. Data analysis

Data obtained from all samples were analyzed using PROC GLM (SAS 9.4 software). Type III tests were used to determine effects of treatments on seed germination while differences between the treatments were assessed using a student t-test with the least square means (LSMEANS) statement, with Tukey’s adjustment for multiple comparisons. Differences were considered significant at *p* ≤ 0.05.

## 3. Results

### 3.1. Mean radicle length

#### 3.1.1. Corn

##### 3.1.1.1. 100 mM NaCl +1.0% EL2006H

**Figure 1:**
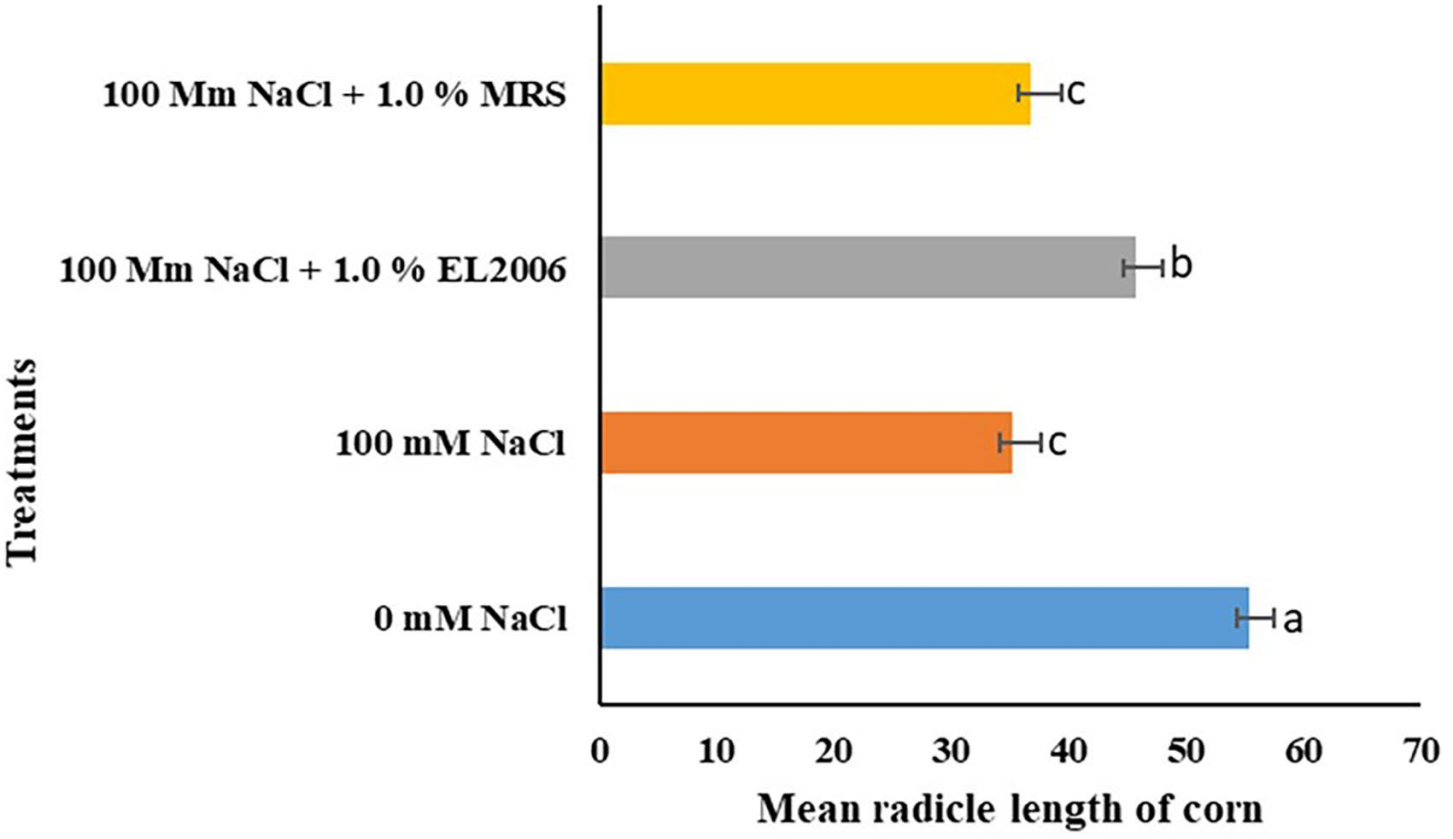
Effect of treatments on radicle length of corn at 1.0% [v/v]. Data represents the mean ± SE (*n* = 80); different letters indicate values determined by Tukey’s multiple mean comparison significantly different at *p* < 0.05. Values with the same letters are not significantly different at *p* < 0.05.

There was a significant effect of *L. helveticus* CFS on radicle length of corn, as shown in Figure 1. One hundred mM NaCl significantly lowered radicle length of corn by 38.58% (*p* < 0.0001) in comparison to the 0 mM NaCl control. Treatment with 100 mM NaCl +1.0% EL2006H significantly increased mean radicle length of corn by 23.04% (*p* < 0.0001). The greatest radicle length was for the 0 mM control (55.38 cm) while the smallest was for the 100 mM NaCl control (35.205 cm), which was also not significantly different from the 100 mM NaCl +1.0% MRS control (36.735 cm).

There was no significant effect of the 0.2% *L. helveticus* CFS on radicle length of corn.

#### 3.1.2. Soybean

**Figure 2:**
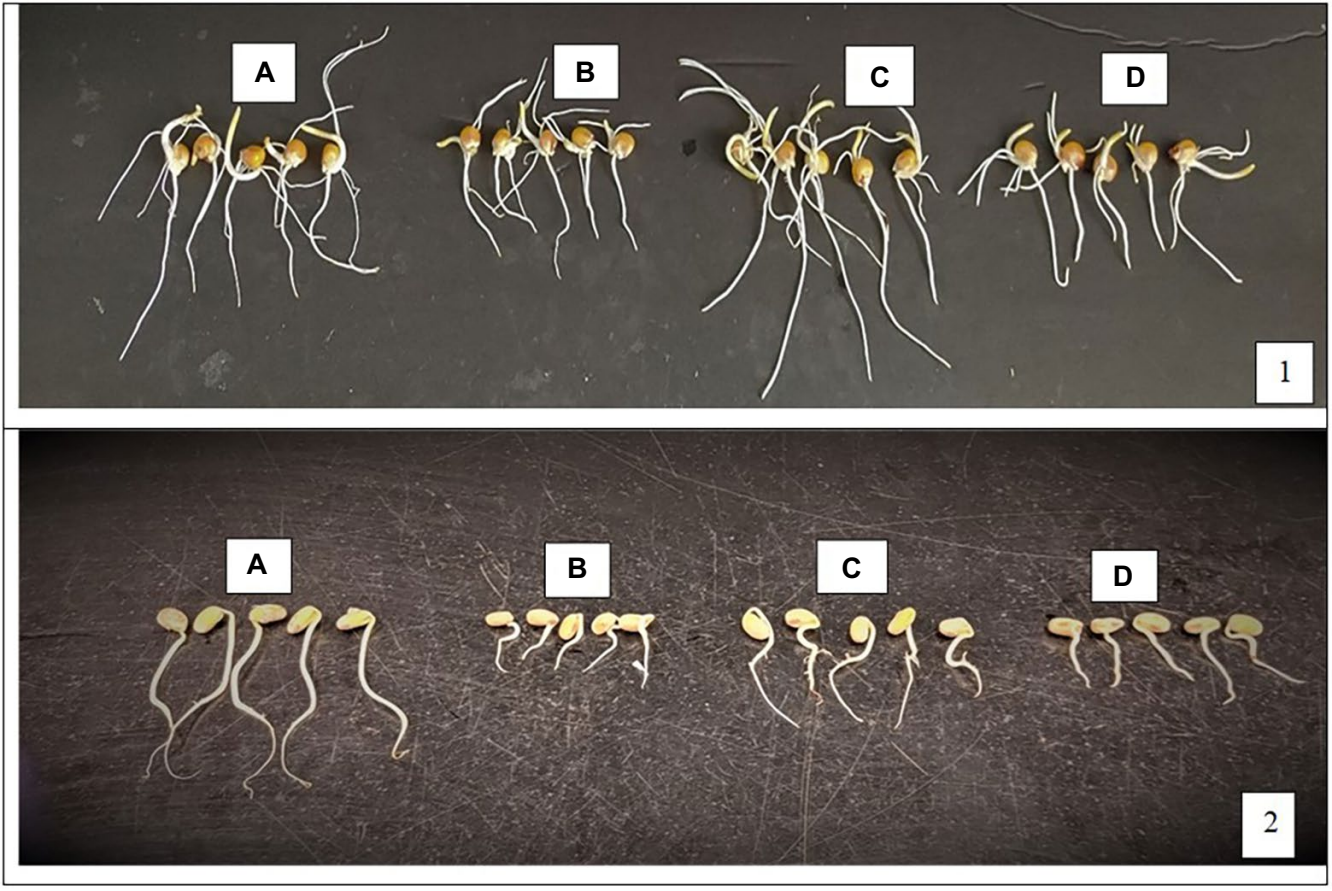
Effect of treatments on germination and radicle length of corn (1) and soybean (2). From left to right: 0 mM NaCl **(A)**, 100 mM NaCl **(B)**, 100 mM NaCl +0.2% EL2006H **(C)** and 100 mM NaCl +0.2% MRS **(D)**.

There was no significant effect of *L. helveticus* CFS on radicle length of soybean at both 0.2 and 1.0% concentrations, as shown in Table 1. Figure 2 shows the effect of treatments on radicle length of corn (1) and soybean (2).

**Table 1:** Effect of treatments on mean radicle length of soybean and corn.

Treatment	Mean radicle length of soybean (cm)	Mean radicle length of corn (cm)
0 mM NaCl	57.98 ± 2.636^a^	49.81 ± 1.879^a^
100 mM NaCl	37.43 ± 1.766^b^	35.15 ± 1.232^b^
100 Mm NaCl +0.2% EL2006	38.05 ± 1.773^b^	38.76 ± 1.193^b^
100 Mm NaCl +0.2% MRS	37.12 ± 2.088^b^	40.26 ± 1.705^b^
0 mM NaCl	63.355 ± 4. 230^a^	55.38 ± 2.084^a^
100 mM NaCl	38.910 ± 2.213^b^	35.205 ± 2.491^c^
100 Mm NaCl +1.0% EL2006	41.195 ± 2.393^b^	45.745 ± 2.233^b^
100 Mm NaCl +1.0% MRS	44.445 ± 3.600^b^	36.735 ± 2.66^c^

Data represents the mean ± SE (*n* = 80); in a column, different letters indicate values determined by Tukey’s multiple mean comparison significantly different at *p* < 0.05. Values with the same letters are not significantly different at *p* < 0.05.

### 3.2. Mean percentage germination

#### 3.2.1. Soybean

##### 3.2.1.1. 100 mM NaCl +0.2% EL2006

At 24 h, there was a significant effect of *L. helveticus* EL2006H CFS on soybean germination. At 48 h, CFS significantly enhanced percentage germination of soybean. Treatment with 100 mM NaCl lowered percentage germination by 50% in comparison to the 0 mM NaCl control. The highest percentage germination was observed in the 0 mM NaCl control (84%) while the lowest was observed in soybean treated with 100 mM NaCl (42%). Percentage germination of soybean treated with 100 mM NaCl +0.2% EL2006H and 100 mM NaCl +0.2% MRS were 75.5 and 67.5%, respectively. The two were significantly higher than that observed for the 100 Mm NaCl control (*p* < 0.0001). In fact, the percentage germination of soybean treated with 100 mM NaCl +0.2% EL2006H CFS was significantly higher than that of the 100 mM NaCl control, by 44.37% (*p* < 0.0001) when treated with CFS and not different from that of the 0 mM NaCl control.

**Figure 3:**
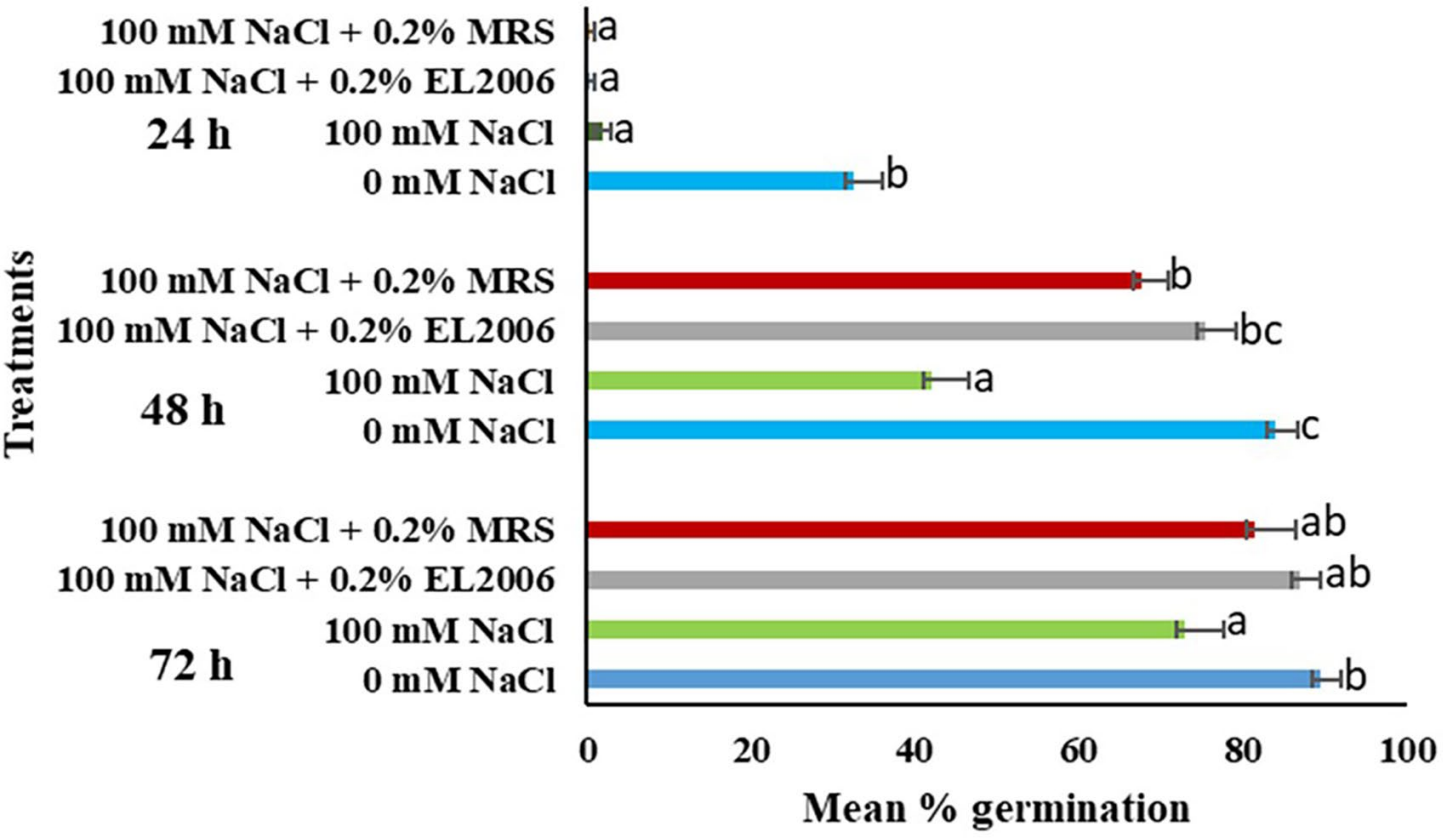
Effect of treatments on mean percentage germination of soybean. Data represents mean ± SE (*n* = 80); different letters indicate values determined by Tukey’s multiple mean comparison significantly different at *p* < 0.05. Values with the same letters are not significantly different at *p* < 0.05.

At 72 h, treatment with microbial CFS did not result in significant differences in the germination of soybean, when compared to the 100 mM NaCl control. However, treatments 100 mM NaCl +0.2% EL2006H increased percentage germination by 16.1, to a percentage not significantly different from the 0 mM NaCl control. Table 2 show the effect of treatments on percentage germination of soybean (Figure 3).

**Table 2:** Effect of treatments on mean percentage germination of soybean, at 72, 48 and 24 h, respectively.

Treatment	Mean percentage germination at 72 h	Mean percentage germination at 48 h	Mean percentage germination at 24 h
0 mM NaCl	89.5 ± 1.697a	86.0 ± 1.835^a^	34.5 ± 2.563^b^
100 mM NaCl	82.5 ± 3.898^a^	66.0 ± 4.834^b^	1.00 ± 0.688^a^
100 mM NaCl +1.0% EL2006	88.5 ± 3.647^a^	70.5 ± 5.452^ab^	0.50 ± 0.500^a^
100 mM NaCl +1.0% MRS	85.0 ± 3.591^a^	74 ± 4.995^ab^	1.00 ± 0.688^a^

Data represents the mean ± SE (*n* = 80); in a column, different letters indicate values determined by Tukey’s multiple mean comparison significantly different at *p* < 0.05. Values with the same letters are not significantly different at *p* < 0.05.

##### 3.2.1.2. 100 mM NaCl +1.0% EL2006

At 24 h, microbial CFS has not significant effect on germination of soybean. At 48 h, soybean treated with 100 mM NaCl +1.0% EL2006H and 100 mM NaCl +1.0% MRS exhibited percentages of 70.5 and 74%, respectively; both values were not significantly different from that of the 0 mM control, and higher, though not significantly different from the percentage germination observed for the 100 mM NaCl control. At 72 h there was no observed significant difference among the percentage germination of the different treatments.

#### 3.2.2. Corn

There was no significant effect of *L. helveticus* CFS on germination percentage of corn at both 0.2 and 1.0% concentrations, as shown in Tables 3, 4.

**Table 3:** Effect of treatments on mean percentage germination of corn at 72, 48 and 24 h, respectively.

Treatment	Mean percentage germination at 72 h ± SEM	Mean percentage germination at 48 h ± SEM	Mean percentage germination at 24 h ± SEM
0 mM NaCl	57.0 ± 3.332^a^	16.0 ± 2.938^a^	0.00 ± 0.000^a^
100 mM NaCl	27.0 ± 4.110^b^	3.5 ± 1.817^b^	0.00 ± 0.000^a^
100 mM NaCl +0.2% EL2006	28.5 ± 3.789^b^	4.0 ± 1.338^b^	0.00 ± 0.000^a^
100 mM NaCl +0.2% MRS	32.0 ± 3.742^b^	0.00 ± 0^b^	0.00 ± 0.000^a^

Data represents the mean ± SE (*n* = 80); in a column, different letters indicate values determined by Tukey’s multiple mean comparison significantly different at *p* < 0.05. Values with the same letters are not significantly different at *p* < 0.05.

**Table 4:** Effect of treatments on mean percentage germination of corn at 72, 48 and 24 h, respectively.

Treatment	Mean percentage germination at 72 h ± SEM	Mean percentage germination at 48 h ± SEM	Mean percentage germination at 24 h ± SEM
0 mM NaCl	71.5 ± 5.144^a^	15.5 ± 1.846^a^	0.00 ± 0.000^a^
100 mM NaCl	30.0 ± 3.770^b^	1.0 ± 0.688^b^	0.00 ± 0.000^a^
100 mM NaCl +1.0% EL2006	34.5 ± 3.515^b^	1.5 ± 0.819^b^	0.00 ± 0.000^a^
100 mM NaCl +1.0% MRS	26.5 ± 3.185^b^	2.0 ± 0.918^b^	0.00 ± 0.000^a^

Data represents the mean ± SE (*n* = 80); in a column, different letters indicate values determined by Tukey’s multiple mean comparison significantly different at *p* < 0.05. Values with the same letters are not significantly different at *p* < 0.05.

#### 3.2.3. Greenhouse experiment

While the above germination experiments were to establish the effects of *L. helveticus* CFS on seed/tuber germination as a possible positive plant growth promoter, the greenhouse experiment with potato was to add more value to the commercial application of the CFS, which is one of the goals of SynAgri/EVL’s mandate and the crop of importance. Hence, experiments were carried out using potato variety goldrush (as per company’s recommendation), to elucidate the effect of *L. helveticus* EL2006H CFS on the growth variables of potato. Results regarding variables varied between treatments.

##### 3.2.3.1. Mean leaf greenness

**Figure 4:**
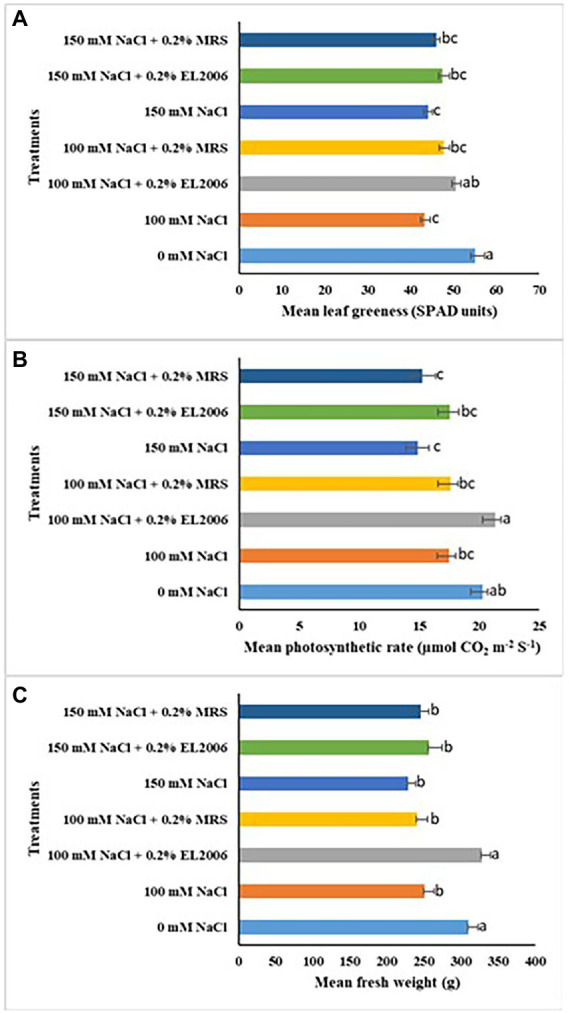
Effect of treatments on leaf greenness **(A)**, photosynthetic rate **(B)** and fresh weight **(C)** of potato. Data represents the mean ± SE (*n* = 48); different letters indicate values determined by Tukey’s multiple mean comparison significantly different at *p* < 0.05. Values with the same letters are not significantly different at *p* < 0.05.

The effect of treatments on mean leaf greenness was significantly different among treatments. Treatment with 100 mM and 150 mM NaCl resulted in 27.014 and 19.88% decreases in mean leaf greenness, respectively, in comparison to the 0 mM NaCl control, the two becoming significantly lower than the later (*p* < 0.0001). Treatment with 100 mM NaCl +0.2% EL2006 resulted in leaf greenness significantly higher than the 100 mM NaCl control by 13.56% (*p* < 0.0001) and not significantly different from the 0 mM NaCl control, as shown in Figure 4. Although not significantly different, it was also higher than the 100 mM NaCl +0.2% MRS control. There was no significant difference between treatment 150 mM NaCl +0.2% EL2006 and its corresponding controls 150 mM NaCl and 150 mM NaCl +0.2% MRS. *L. helveticus* EL2006H CFS enhanced leaf greenness in potato treated with 100 mM NaCl but not 150 mM NaCl.

##### 3.2.3.2. Mean photosynthetic rate

There was a significant effect of *L. helveticus* CFS on mean photosynthetic rate. as shown in Figure 4. Treatment with 100 and 150 mM NaCl resulted in 13.76 and 26.6% decreases in photosynthetic rate, respectively, in comparison to the 0 mM NaCl control. Treatment with 100 mM NaCl +0.2% EL2006 resulted in the highest photosynthetic rate (21.00 μmol CO_2_ m^−2^ S^−1^), significantly higher than that of the 100 mM NaCl control, by 17.97% (*p* < 0.0001), and higher but not significantly different from the 0 mM NaCl control, as shown in Figure 4. The lowest photosynthetic rate was observed in potato treated with 150 mM NaCl (14.863 μmol CO_2_ m^−2^ S^−1^).

##### 3.2.3.3. Mean fresh weight

**Figure 5:**
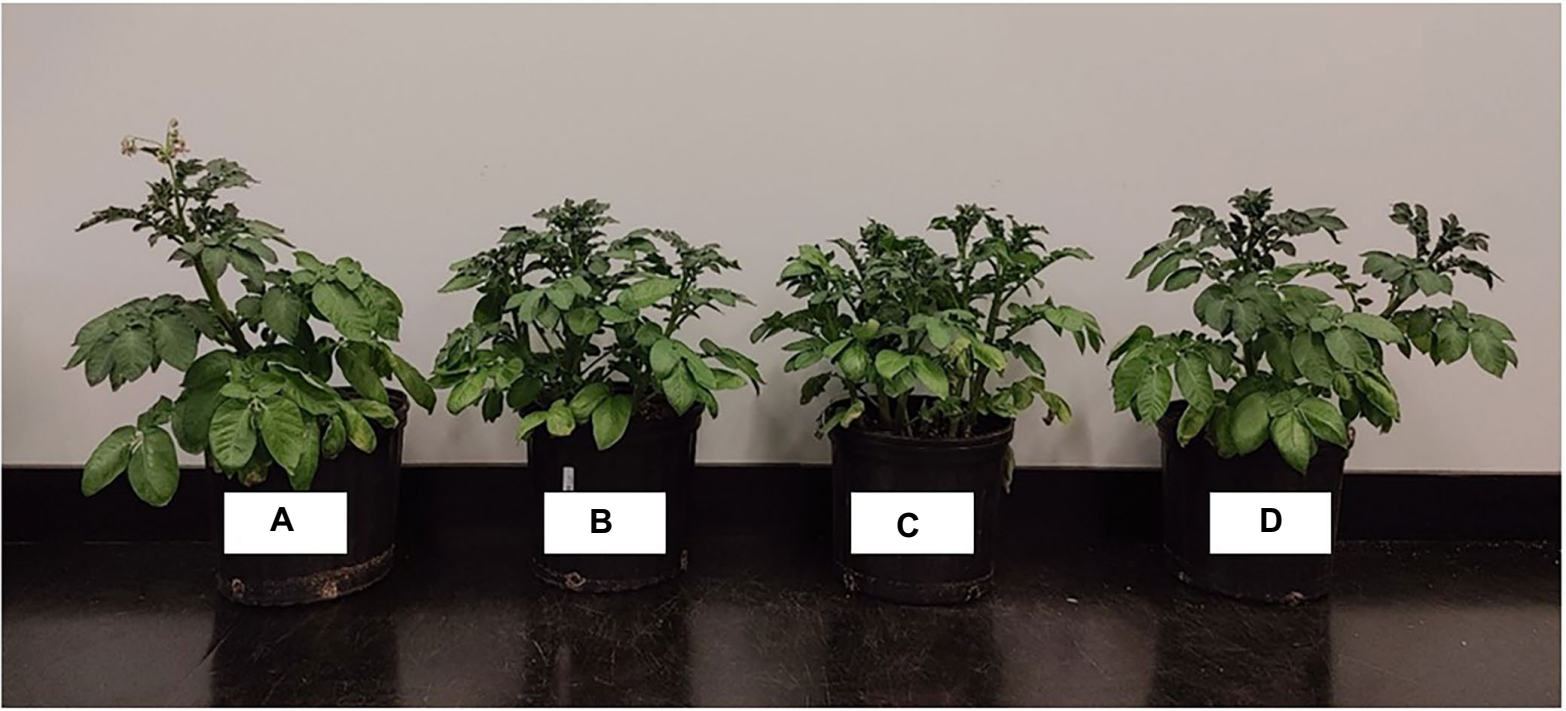
Potato treated with 0 mM NaCl **(A)**, 100 mM NaCl **(B)**, 100 mM NaCl +0.2% MRS **(C)** and 100 mM NaCl +0.2% EL2006H **(D)**.

There was a significant effect of *L. helveticus* CFS on fresh weight of potato. One hundred mM NaCl and 150 mM NaCl lowered fresh weight of potato by 19.62 and 23.61%, respectively, in comparison to the fresh weight observed for the 0 mM NaCl control. Treatment with 100 mM NaCl +0.2% EL2006 resulted in potato with fresh weight higher but not significantly different from the 0 mM control. It was also significantly higher than the fresh weight of potato treated with 100 mM NaCl +0.2% MRS control (Figure 5).

There was no significant effect of *L. helveticus* CFS on leaf area and plant height of potato, as shown in Table 5.

**Table 5:** Effect of treatments on selected growth variables of potato.

Treatment	Height gain (cm)	Leaf area (cm^2^)
0 mM NaCl	37.275 ± 7.531^a^	2851.261 ± 158.736^a^
100 mM NaCl	32.263 ± 5.739^a^	2951.652 ± 180.931^a^
100 mM NaCl +0.2% EL2006	34.838 ± 6.937^a^	3358.082 ± 156.557^a^
100 mM NaCl +0.2% MRS	33.113 ± 6.129^a^	2943.797 ± 234.763^a^
150 mM NaCl	38.9 ± 2.282^a^	3045.75 ± 157.209^a^
150 mM NaCl +0.2% EL2006	40.825 ± 1.723^a^	2960.841 ± 87.894^a^
150 mM NaCl +0.2% MRS	40.063 ± 2.233^a^	3076.615 ± 211.951^a^

Data represents the mean ± SE (*n* = 48); in a column, different letters indicate values determined by Tukey’s multiple mean comparison significantly different at *p* < 0.05. Values with the same letters are not significantly different at *p* < 0.05.

## 4. Discussion

There is a continuous need to increase food production to quantities sufficient to feed the growing human population, without compromising quality, using sustainable and environmentally friendly approaches such as plant growth promoting microorganisms (PGPM) and their derived compounds (Lyu et al., 2020; Shah et al., 2021). NaCl stress is a major global constraint to food production (Naamala and Smith, 2021b). Plant growth promoting microorganisms (PGPM), or their derived compounds have been reported to enhance plant growth under saline conditions (Schwinghamer et al., 2016; Subramanian et al., 2016a,b; Ilangumaran et al., 2021; Naamala et al., 2022). However, use of microbial CFS as plant growth biostimulants is less explored, with just a handful of publications available (Tewari et al., 2020; Shah et al., 2022; Naamala et al., 2022). It is possibile that CFS could enhance plant growth because microbes exude metabolites into their growth media in response to various signals, such as those related to biotic or abiotic stress (Subramanian et al., 2021). Among the exuded metabolites are some that possess phyto-stimulation properties, such as IAA, LCO and thuricin17 (Prithiviraj et al., 2003; Mohite, 2013; Subramanian et al., 2016a,b; Antar et al., 2021). In the laboratory setting, such signals/metabolites are exuded into the microbe’s growth medium, which when filtered of microbial cells, will still contain the metabolites that can then enhance plant growth (Gray et al., 2006a). Consequently, the possible modes of action through CFS could enhance plant growth include presence of phytohormones such as jasmonic acid; presence of enzymes such as ACC deaminase; presence of osmoprotectants such as proline and presence of volatile organic compounds and exopolysaccharides, all of which may function to mitigate osmotic, oxidative and ionic stress associated with salinity (Forni et al., 2017; Khan et al., 2019; Cappellari and Banchio, 2020; Kumar et al., 2020; Fincheira et al., 2021; Lopes et al., 2021). There is limited publication on the role of members of the genus *Lactobacillus* and/or their CFS as plant growth biostimulants (Hamed et al., 2011; Lamont et al., 2017; Msimbira et al., 2022). The modes of action through which members of the genus *Lactobacillus* and their CFSs enhance plant growth are not fully understood (Lamont et al., 2017). However, plant growth promotion by LAB species has been attributed to production of metabolites such as IAA and siderophores (Omer et al., 2010; Mohite, 2013; Shrestha et al., 2014; Limanska N.V. et al., 2015; Limanska N. et al., 2015), and solubilisation of phosphorus (Shrestha et al., 2014), among other mechanisms. In general, PGPM and or their derived compounds can mitigate salinity stress by employing one or more of the following mechanisms: Production of antioxidants, production of enzymes such as *ACC* deaminase, production of exopolysaccharides, inducing systemic resistance in plants (Shrivastava and Kumar, 2015; Amna et al., 2019) and production of microbe-to-plant signal compounds (Backer et al., 2018).

The current study focused on the ability of CFS obtained from *L. helveticus* EL2006H to enhance growth of three crop species: corn, soybean and potato, exposed to NaCl stress, under controlled conditions. Results of the study highlight the role CFS concentration, NaCl level, crop species and growth level play in the effectiveness and efficacy of CFS as plant growth biostimulants.

The effect of CFS on mean radicle length varied among crop species, concentration of the CFS, and level of NaCl in the treatment. For instance, treatment with 100 mM NaCl +1.0% EL2006H resulted in a significant increase in corn radicle length but not soybean, suggesting crop specific responses. Although some PGPM, such as some *Rhizobium* species, can be promiscuous, enhancing growth in a wide range of crop species, others are host specific, enhancing growth of just one or two crop species (Lyu et al., 2020). PGPM and their host plants communicate through signals, which vary depending on the host plant needs (Antar et al., 2021). Sometimes, such signals will limit the host range of a particular PGPM. However, such PGPM can produce metabolites that enhance growth in a wide range of crops. For instance, LCO, can enhance growth of non-legumes although it is produced by *B. japonicum*, and plays a major role in nodulation of soybean. It is such advantages that make CFS and plant derived compounds relevant in PGPM technology (Naamala and Smith, 2020). Even then, there is not yet a single microbial derived compound or PGPM that enhances growth of all crop species. It is possible that the varied responses to CFS observed in corn, soybean and potato are in part due to variation in ways through which the three crops perceive and respond to the bioactive signals in the CFS. In another study, we also observed variation in soybean and corn responses to *B. amyloliquefaciens* CFS (Naamala et al., 2022). The *B. japonicum* derived LCO also exhibited variation in its effect on corn and soybean (Souleimanov et al., 2002). At a lower concentration, variations were observed in the response of canola varieties treated with LCO (Schwinghamer et al., 2015).

Lowering CFS concentration from 1.0% (v/v) to 0.2% EL2006H CFS resulted in no significant effect on both corn and soybean mean radicle length. This seems to suggest that the concentration and quantity of CFS applied to a plant is vital in determining efficacy and effectiveness of the applied CFS in enhancing plant growth. In this case, especially for corn, higher concentrations resulting in more effective results than lesser concentrations. The same cannot be said about soybean. Its possible that in soybean, perhaps minute quantities of the supernatant were enough to enhance growth. For example, Msimbira et al. (2022) observed variation in germination of corn, in response to *Bacillus subtilis* CFS, where a concentration of 0.1% (v/v) yielded better results than higher concentrations of 0.2, 0.4 and 1.0%. It should also be noted that high concentrations may sometimes inhibit growth of the crop in question (Naamala et al., 2021b), although there is no universal description of how much is sufficient and this could vary among crop species. Published studies on *L. helveticus* CFS as a plant biostimulant are currently limited to a study by Msimbira et al. (2022). In our previous study on *B. amyloliquefaciens* EB2003 CFS, we observed the effect of CFS concentration on its effectiveness in enhancing germination and radicle length of corn and soybean (Naamala et al., 2022). Studies on other members of the genus *Lactobacillus* have also reported concentration as a major determinant of effectiveness and efficacy in plant growth promotion. For instance, radish plants responded differently to varying concentrations of *L. plantarum* (Higa and Kinjo, 1991).

Results on mean percentage germination varied between corn and soybean at the two CFS concentrations and three different time frames studied. For instance, in soybean, following treatment with 100 mM NaCl +0.2% EL2006H, greatest significance was observed at 48 h, where percentage germination of soybean was not only significantly higher than that of the 100 mM NaCl control, but was also not significantly different from that of the unstressed 0 mM NaCl control. At 24 h, there was no observed difference among the effects of treatments for percentage germination. It is however possible that the CFS was already working on the physio-chemical properties of the seed to mitigate the effect of NaCl on the plant, hence the higher percentage germination observed at 48 h. The plant also naturally attempts to put in place a defence against stress, but can be slower, which could explain why at 72 h, there was no significant difference in the percentage germination of all the treatments on soybean. Therefore, it seems likely that at 48 h, CFS mitigated delays in germination due to 100 mM stress, resulting in percentage germination levels higher than the stressed control and not significantly different from the unstressed control, which is desirable because slow germination exposes seed to attack by pathogens in the soil, among other disadvantages. Similar results were obtained by Msimbira et al. (2022) who observed variation in the effect of EL2006H CFS on germination of corn and tomato across time. Naamala et al. (2022) also observed variation in effect of *B. amyloliquefaciens* CFS among 24, 48 and 72 h sampling times. In corn however, there was no significant effect of the EL2006H CFS on mean percentage germination at all time intervals. The mean percentage germination of corn was significantly lower than the unstressed control at both 48 and 72 h. This again takes us back to the effect of crop species on effectiveness of the CFS. The effect of PGPM CFS and other PGPM derived compounds on seed germination has been reported by other researchers (Tallapragada et al., 2015; Subramanian et al., 2016a,b; Shah et al., 2022). *Devosia* sp. CFS enhanced germination of canola and soybean under NaCl stressed and optimal conditions (Shah et al., 2022).

Increasing concentration from 0.2 to 1.0% of CFS yielded less desirable results in soybean as there was no significant difference between mean percentage germination of soybean treated with 100 mM NaCl +0.2% EL2006H and 100 mM NaCl, at all measurement times, although the percentage germination of the latter was not significantly different from that observed in the 0 mM control at 48 h. Results seem to suggest that when it comes to mean percentage germination, lesser quantities of the CFS are required to mitigate the effect of NaCl stress on germination of soybean. In corn, CFS had no significant effect on mean percentage germination, with the unstressed control still significantly higher at both 48 and 72 h. Shah et al. also observed variations in the germination of canola and soybean treated with a range of concentrations of *Devosia* sp. CFS (Shah et al., 2022).

In the potato experiment, results exhibited effects of stress level on effectiveness of EL2006H CFS. The supernatant enhanced some growth variables but not all. Specifically, CFS enhanced fresh weight, photosynthetic rate and leaf greenness but not leaf area and plant height. Among the variables enhanced, levels varied across NaCl levels. Better results of the CFS were observed in potato treated with 100 mM NaCl than 150 mM NaCl. This implies that increasing NaCl level by 50 mM reduced effectiveness of the CFS. This is not surprising as its possible that the potential bioactive substance was less effective at 150 mM NaCl so that it/they could not function as efficiently as they would at lower concentrations, or, at 150 mM NaCl, potato plant cell components could have been damaged (Ilangumaran et al., 2021) to a point where even the CFS could not fully mitigate these effects on growth. The effect of NaCl level on the effectiveness of CFS was also observed by Naamala et al., (2022) on percentage germination and radicle length of soybean and corn treated with different concentrations of NaCl. The CFS enhanced radicle length in soybean not stressed with NaCl but not in soybean exposed to NaCl while the reverse was true for corn (Naamala et al., 2022). Subramanian et al. (2016a) also observed that LCO enhanced germination of soybean exposed to 100 mM NaCl but not higher NaCl concentrations of 150 mM NaCl and 175 mM NaCl. Shah et al. observed significant increases in germination of canola and soybean treated with *Devosia* sp. CFS (Shah et al., 2022).

The ability of PGPM, LAB included, to enhance plant growth can be affected by plant growth stage and growth variables, in which case a microbial strain or its CFS can enhance plant growth at a certain plant growth stage and not at another or enhance growth of one variable but not the other. For instance, under field conditions, Shrestha et al. (2014) observed an increase in growth of pepper plants treated with each of the three LAB they were studying, 1 week after transplanting. However, after that stage, only one strain of the three was able to enhance plant height. This, in a way, points out the complexity of depending on the plant-microbe interactions for plant growth stimulation.

Published research on *L. helveticus* as a plant growth biostimulant remains minimal. However, other members of the genus have been reported to enhance plant growth. For example, *L. plantarum* exhibited antimicrobial activity against *Fusarium* spp. in agar plate assays and a consortium consisting of the same and *B. amyloliquefaciens* reduced severity of *Fusarium* spp. in wheat (Baffoni et al., 2015). *L. plantarum* ONU 12 expressed antimicrobial properties in carrot, kalanchoe and grapes exposed to *Agrobacterium tumefaciens*, protection ranging from 72.7 to 100% of wounded kalanchoe tissues, depending on mode of application (Limanska N. et al., 2015). LAB species KLF01, KLC02 and KPD03 had antagonistic effects against *Xanthomonas campestris pv. vesicatoria* (Shrestha et al., 2014) while strains LB-1, LB-2 and LB-3 increased total fresh weight of tomato plants by 348, 260, and 390%, respectively (Hamed et al., 2011). LAB species identified as KLF01, KLC02 and KPD03 were reported to enhance chlorophyll content in pepper (Shrestha et al., 2014). The same strains, except KLC02, were reported to enhance shoot length in pepper (Shrestha et al., 2014). Recently, research on PGPM has extended to their CFSs, attempting to elucidate whether it can enhance plant growth in the absence of the microbial cell. The ability of *L. helveticus* CFS at varying pH levels to enhance plant growth was recently reported (Msimbira et al., 2022). The effectiveness and efficacy of PGPM or their CFS, including LAB is dependent on several soil, plant and microbe factors, such soil conditions and plant species. A PGPM can enhance growth of one crop species but not another or enhance growth under certain soil conditions, such as under stressed conditions but not under optimal conditions.

At this stage we do not know whether the effect of CFS on the different variables is from a single bioactive compound or more than one; both scenarios are possible. As mentioned earlier, knowledge on the mechanisms employed by members of the genus *Lactobacillus* and or their CFSs to enhance plant growth remain poorly understood.

## 5. Conclusion

Based on the results of this study, it seems likely that *L. helveticus* EL2006H exudes into its growth media substances which enhance radicle length in corn, mean percentage germination in soybean and photosynthetic rate, greenness and mean fresh weight in potato. However, the effect varies depending on crop species, concentration of CFS and level of NaCl stress. *L. helveticus* CFS concentration of 0.2 and 1.0% were more effective at enhancing radicle length in corn and percentage germination in soybean, respectively. One hundred and fifty mM NaCl lowered the effectiveness of the 0.2% concentration in enhancing leaf greenness, mean photosynthetic rate and mean fresh weight of potato. Findings of this study can be used as a basis to further study of *Lactobacillus* CFSs and possibly identify the bioactive substances therein. Findings of this study are promising in the field of microbial inoculants where new ways of improving efficacy and effectiveness of the technology, especially under field conditions, are constantly sought. However, further studies need to be done, especially under field conditions, with trials on different crop species and varying soil conditions.

## Data availability statement

The original contributions presented in the study are included in the article/supplementary material, further inquiries can be directed to the corresponding author.

## Author contributions

JN set up the experiment and wrote the manuscript. LM helped with data collection and experimental set up. SS advised on scientific approach and provided background knowledge. DS provided funding, the intellectual context, and extensive editorial input and guided in scientific knowledge. All authors contributed to the article and approved the submitted version.

## Funding

This work was funded through a grant from Consortium de recherche et innovations en bioprocédés industriels au Québec, number CRIBIQ 2017-034-C30, with support from synagri and EVL inc.

## Conflict of interest

The authors declare that the research was conducted in the absence of any commercial or financial relationships that could be construed as a potential conflict of interest.

## Publisher’s note

All claims expressed in this article are solely those of the authors and do not necessarily represent those of their affiliated organizations, or those of the publisher, the editors and the reviewers. Any product that may be evaluated in this article, or claim that may be made by its manufacturer, is not guaranteed or endorsed by the publisher.
